# HCMV-Mediated Interference of Bortezomib-Induced Apoptosis in Colon Carcinoma Cell Line Caco-2

**DOI:** 10.3390/v13010083

**Published:** 2021-01-09

**Authors:** Heike Härtel, Janine Theiß, Mohammed O. Abdelaziz, Martin J. Raftery, Gabriele Pecher, Elke Bogner

**Affiliations:** 1 Institute of Virology, Charité-Universitätsmedizin-Berlin, 10117 Berlin, Germany; heike.haertel@ymail.com (H.H.); TheissJanine@web.de (J.T.); mohammed.yassen@charite.de (M.O.A.); 2 Medical Clinic of Hematology, Oncology and Tumorimmunology, CCM, Charité-Universitätsmedizin-Berlin, 10117 Berlin, Germany; martin.raftery@charite.de (M.J.R.); gabriele.pecher@charite.de (G.P.)

**Keywords:** human cytomegalovirus, Caco-2 cells, Bortezomib, apoptosis, necroptosis, co-culture

## Abstract

Human cytomegalovirus (HCMV) has been implicated in the development of human malignancies, for instance in colon cancer. Proteasome inhibitors were developed for cancer therapy and have also been shown to influence HCMV infection. The aim of this study was to investigate if proteasome inhibitors have therapeutic potential for colon carcinoma and how this is influenced by HCMV infection. We show by immunofluorescence and flow cytometry that the colon carcinoma cell line Caco-2 is susceptible to HCMV infection. Growth curve analysis as well as protein expression kinetics and quantitative genome analysis further confirm these results. HCMV has an anti-apoptotic effect on Caco-2 cells by inhibiting very early events of the apoptosis cascade. Further investigations showed that HCMV stabilizes the membrane potential of the mitochondria, which is typically lost very early during apoptosis. This stabilization is resistant to proteasome inhibitor Bortezomib treatment, allowing HCMV-infected cells to survive apoptotic signals. Our findings indicate a possible role of proteasome inhibitors in colon carcinoma therapy.

## 1. Introduction

Human cytomegalovirus (HCMV), one of nine human herpesviruses, can cause serious illness in neonates as well as in immunocompromised adults [1,2,3]. Although HCMV is not thought to be a classical oncovirus, recent reports provide evidence that HCMV is also associated with human malignancies. Viral antigen as well as nucleic acids have been detected in large proportions of malignant tumors: colon cancers, prostate cancer, glioblastomas, medullablastomas, and breast cancer [4,5,6,7,8]. HCMV DNA was detected by PCR in 42.3% of the tumor specimens, while only 5.6% samples of adjacent non-neoplastic tissue were positive for HCMV [9]. Quantitative real-time PCR in 54 sample pairs revealed significantly higher viral copies in the tumor specimens than the adjacent non-neoplastic tissue specimens [9]. Colorectal cancer (CRC) is a leading cause of cancer death worldwide [10]. The presence of HCMV in CRC is associated with poorer outcome in elderly patients [11]. The results suggest that HCMV may influence the outcome of CRC in an age-dependent manner and possibly has a dual oncomodulatory effect [11]. The presence of HCMV in tumors does not correlate with the serological or viremic status of CRC patients [12]. HCMV reactivation in CRC patients is possibly due to virus-cancer interactions in the CRC tumor microenvironment [13], resulting in active viral gene transcription in the tumor but not in the blood of CRC patients. It has been suggested that HCMV leads to a dysregulation of multiple cellular pathways involved in oncogenesis [14,15,16]. In the present study, we analyzed the effects of HCMV infection of the colon carcinoma cell line Caco-2 and its interaction with the proteasome inhibitor Bortezomib. Bortezomib targets specificity protein (Sp) transcription factors in cancer cells [17]. In addition, Bortezomib induces G2-M arrest in human colon cancer cells through ROS-inducible phosphorylation of ATM-CHK1 [18]. Some reports have shown that tegument proteins prevent degradation either by suppression of ISGylation (pUL25) [19], inactivation of the DNA sensor cGas (pp65) [20] or –STING degradation (IE2) [21]. Interestingly, HCMV UL35 downregulates BclAF1 by proteasomal degradation [22]. BclAF1 induces apoptosis by binding to anti-apoptotic Bcl proteins such as Bcl-2 and Bcl-xl, and Bortezomib induces apoptosis in part by increasing pro-apoptotic BH3 proteins [23]. In contrast, anti-apoptotic HCMV proteins inhibit different pathways of apoptosis. HCMV protein vMIA (pUL37 × 1), the viral mitochondria-localized inhibitor of apoptosis, inhibit the two pro-apoptotic effector proteins Bax and Bak and lead to proteasomal degradation. Both proteins are localized at the mitochondria outer membrane and induce permeabilization of this membrane during apoptosis [24,25]. HCMV pUL36 known as the viral inhibitor of caspase-8 activation (vICA) prevents proapoptotic signaling by binding to caspase-8 [26]. Another protein that blocks apoptosis is pUL38 that antagonizes cellular stress responses [27].

One important cellular pathway involved in the pathogenesis of colorectal cancer is the NF-ĸB regulated proteasome system [28]. Clinical data provide evidence that the proteasome plays an important role in maintaining the immortal phenotype of myeloma cells. Furthermore, similar effects have been shown in solid organ cancers [29]. Cancer cells are exposed to protein overload and needs the proteasome system. Levin et al. [30] provide evidence that during tumor progression the 26S proteasome assembly was enhanced. Thus, enhanced CRC–associated proteasome assembly is a prerequisite to manage protein overload. In this regard, Bortezomib inhibition of the 26S proteasome leads to growth inhibition and apoptosis [31,32]. One mechanism will be the destruction of the major stress response of the cancer cells. Furthermore, Bortezomib is the first proteasome inhibitor approved for use at different points in the treatment of multiple myeloma [33]. These insights inspired us to analyze the effect of HCMV in Caco-2 cells and its effect on Bortezomib in these cells.

## 2. Materials and Methods

### 2.1. Cells and Virus

Human colon adenocarcinoma cell line Caco-2 (ECACC, Porton Down, England) and human embryonic lung fibroblasts Fi301 (HELF Fi301, authenticated by Deutsche Sammlung von Mikroorganismen und Zellkulturen; DSMZ, Braunschweig, Germany) were grown in Dulbecco’s minimal essential medium (DMEM) supplemented with 10% fetal bovine serum (FBS), 2 mM glutamine and gentamycin (60 µg/mL). Caco-2 cells and HELF Fi I301 at passages 9 to 25 or 10 to 15 were used for infections. Experiments were carried out with confluent cell monolayers.

Infection of Caco-2 or HELF Fi301cells with a clinical isolate of HCMV (GCV-res) or TB40/E-pp150EGFP (kindly provided by C. Sinzger, Ulm Medical Center, Institute of Virology [34]) was carried out as previously described [35]. To enhance infectivity, Caco-2 cells were (i) cultured for 24 h in medium supplemented with 2% FBS, (ii) infected with HCMV at a MOI 10, and (iii) centrifuged for 45 min during the absorption of the virus. Proteasome inhibitor was added after adsorption at the indicated time points.

The HCMV clinical isolate used is resistant against Ganciclovir, because it carries the substitution M460I and M406V in HCMV UL97. It was received from K. Hamprecht (Institute of Medical Virology, Tübingen, Germany) at passage 4 and was used at passage 9, respectively. Differentiated Caco-2 cells can only be infected from the basolateral side [36]. Therefore, the tight junction formations between the cells were temporarily blocked by pretreatment with 100 µM EGTA for 1 h). The effects of EGTA are fully reversible upon removal of the compound [36,37].

### 2.2. Proteasome Inhibitors

Stocks of 10 mM Bortezomib (Selleck Chemicals LLC, Houston, MA, USA) were prepared in 95% (*w*/*v*) dimethyl sulfoxide (DMSO) and stored at −20° C. For further applications the inhibitor was diluted in medium.

### 2.3. Immunofluorescence

Caco-2 cells or HELF Fi301 (6.5 × 10^4^) were seeded on cover slips and mock-infected or infected with HCMV isolate or HCMV TB40/E-pp150EGFP at a MOI of 10. At the appropriate time point cells were fixed with 3% paraformaldehyde as described previously [35]. Detection of HCMV IE1/IE2, pUL44 and—by infection with the HCMV isolate- pp28 was carried out with the antibodies mAb CCH2/DDG9 (Dako-Agilent Technologies, Santa Clara, CA, USA), mAb CH16 (Santa Cruz Biotechnology, Heidelberg, Germany) and mAb 5C3 (Santa Cruz Biotechnology, Heidelberg, Germany) for 45 min at room temperature prior to further incubation for 45 min with Cy5-labeled goat anti-mouse F(ab’)_2_ fragments. After staining, samples were mounted in Fluoromount-G (Thermo Fisher Scientific, Waltham, MA, USA) with 2.5% (*w*/*v*) 1,4-Diazabicyclo [2.2.2] octan and examined under the confocal laser scanning microscope Eclipse TiA1/A1R (Nikon Instruments Europe BV, Amsterdam, The Netherlands). Images were captured with NIS-Elements ARprogram (Nikon Instruments Europe BV). For kinetic analysis with HCMV TB40/E-pp150EGFP mock-infected or infected cells were fixed with 4% paraformaldehyde at appropriate time point. Detection of HCMV IE1/2 and pUL44 was carried out with the antibodies CCH2/DDG9 (Dako) and CH16 (Santa Cruz Biotechnology) for 30 min at room temperature followed by incubation for 20 min with DyLight-549 goat anti-mouse IgG H+L (Jackson ImmunoResearch Europe Ltd, Ely, UK) and Alexa Fluor 647 donkey anti-mouse IgG F(ab′)_2_ fragments (Thermo Fisher Scientific, Waltham, MA, USA). Additional expression of pp150 was determined by EGFP signal. The kinetic analysis were examined under the confocal laser scanning microscope TCS SPE (Leica Camera AG, Wetzlar, Germany). Images were captured with LAS X program (Leica Camera AG, Wetzlar, Germany).

### 2.4. Quantitative Analysis of Infected Cells by Flow Cytometry

Caco-2 cells were infected for 7 days, harvested and the degree of infection was determined by staining with antibodies against IE1/2 (CCH2, DDG9 Dako) and pp28 (5C3; Santa Cruz Biotechnology) or for kinetic analysis IE1/2 and pUL44 (CH16. Santa Cruz) and cytofluorimetric analysis. Briefly, the cells were trypsinised, washed with ice-cold buffer 1 (PBS, 1% BSA, 0.2% sodium azide), resuspended in buffer 1 with 99% EtOH, incubated for 20 min at 4 °C, and then washed again prior to incubation with the first mAb (1:150) for 1 h. The cells were then washed and incubated with secondary Alexa-Fluor-488-conjugated donkey anti-mouse-IgG (Jackson ImmunoResearch Laboratories; 1:400) for 1 h. After the final staining step, cells were washed in ice-cold buffer 1 and then resuspended in 100 µL PBS. Flow cytometry was performed on a FACSCalibur (BD Biosciences-EU, Eysins, Switzerland) or on a BD FACSLyric (for the kinetic experiments). The images were analyzed with BD CellQuest Pro or BD FACSuite V1.2.1 software package (BD bioscienes-EU, Eysins, Switzerland). The generation of bar charts was performed by using the software GraphPadPrism V 8.4.1 (GraphPad Software, San Diego, CA, USA).

### 2.5. Cell Proliferation Assay

Caco-2 cells (1.45 × 10^4^) were seeded in 96-well plates. Confluent cells were incubated with 0.1, 0.2, 0.3, 0.4, 0.5, 0.6, 0.7, 0.8, 0.9, and 1.0 mM of Bortezomib in a final volume of 100 µL for 24 h at 37 °C. Cytotoxicity (50% cytotoxic concentration, CC_50_) profiling of bortezomib was determined by the use of Cell Proliferation Kit II (XTT, Roche, Mannheim, Germany) as recommended by the manufacturer. The absorbance of 492 nm with a reference wavelength of 650 nm was measured. The assay is based on the cleavage of the yellow tetrazolium salt XTT and the formation of the orange formazan dye by metabolic active cells. An increase in number of living cells directly correlates to the amount of orange formazan formed.

In addition, cytotoxicity produced in Caco-2 cells was determined by microscopic inspection of cells not affected by the virus used in HCMV plaque assays similar to Turk et al. [38].

### 2.6. Plaque Reduction Assay

Cells (6.5 × 10^4^) were seeded in 24-well plates and infected with HCMV isolate. After 1.5 h p.i. the inoculum was discarded, the cells were washed for three times with PBS and overlaid with 2 mL methylcellulose (Methocel MC, Fluka Analytical, München, Germany) containing DMEM with 10% FBS and 0.1–12.0 nM Bortezomib. After incubation for seven days at 37 °C, the cells were fixed using an -ethanol-acetone composition (95:5) and stained using an IE1/2 antibody (CCH2, DDG9 Dako) followed by treatment with the AEC (3-Amino-9-ethylcarbazole) staining kit (Sigma-Aldrich, Deisenhofen, Germany) as recommended by the manufacturer. Plaques were counted by the use of a light microscope (Axiovert 10, Carl Zeiss Microscopy GmbH, Jena, Germany). Compound effects were calculated by comparing compound-treated cells versus untreated cells.

### 2.7. Yield Assay

Confluent Caco-2 cells (6.7 × 10^4^) grown in 24-well plates were infected with HCMV isolate or isolate inactivated by UV-irradiation (312 nm on ice for 10 min (UV Transilluminator TI 5, Biometra), MOI 10). After 1 h, the inoculation was discarded and the cells were washed with PBS pH3.0 for 3 min followed by washing with PBS pH 7.4 for 5 min. At 1, 2, 3, 4, 5, 6, and 7 d post infection, supernatants and cells were harvested and frozen at −80 °C. After collection of all time points, supernatants, UV-inactivated supernatants, cells and UV-inactivated cells were transferred to HELF Fi301 grown on 12-well plates, overlaid with methylcellulose and titers were determined 7 d p.i. by plaque assay. Fixation and staining were as described by plaque reduction assay.

### 2.8. Real-Time Quantitative PCR

Caco-2 cells (6.7 × 10^4^) were seeded in 24-well plates and infected with HCMV isolate. After one hour, the inoculum was discarded and the cells were washed for three times with PBS. At 36, 96, and 168 h p.i. the cells were lysate with SDS buffer followed by DNA isolation with phenol-chloroform treatment and ethanol precipitation. Amounts of viral DNA were determined by quantification of real-time PCR. Oligonucleotides for real-time PCR amplifying pp150 were: pp150-fwd 5′-TCCCTTCAGGATGCCTACGA-3′ and pp150-rev: 5′-TAATCGGACGACGGTGTTGT-3′ Real-time PCR was performed using 500 ng/µL of Template DNA and 10 µL 5 × Phusion Green HF buffer (Thermo Fisher Scientific, Henningsdorf, Germany; including 7.5 mM MgCl_2_), 0.02 U/µL Phusion DNA Polymerase, 200 µM for each dNTPs, 0.5 µM for each pp150 primer in a final volume of 50 µL. After activation of the polymerase at 98 °C for 2 min, 45 cycles of amplification (98 °C for 15 s, 64 °C for 15 s, and 72 °C for 10 s), were performed in a qTOWER^3^ (Analytik Jena AG). In the assay we used one negative (mock-infected cells) and one positive control (HCMV TB40/E BAC4 DNA). The probes were normalized against TB40/E BAC4 DNA.

### 2.9. PAGE and Western Blot Analysis

Caco-2 cells (6.7 × 10^4^) were seeded in 24-well plates and infected with HCMV isolate. After one hour the inoculum was discarded and the cells were washed for three times with PBS. At 0, 36, 96, and 168 h p.i. cells extracts (pooled triplicates) were solubilized in 4 × sample buffer (4% (*v/v*) ß-mercaptoethanol, 0.01% (*w*/*v*) bromophenol blue, 4% (*w*/*v*) glycerol, 4% (*w*/*v*) SDS, 0.2 M Tris-HCl (pH 6.8)) prior to separation on 10% (*w*/*v*) SDS-PAGE. Proteins were transferred to nitrocellulose sheets and subjected to Western blot analysis as described previously [35]. The antibody CCH2/DDG9 (Dako-Agilent Technologies; 1:500) specific for IE1/2 were used as the primary antibodies. For detection of primary antibody binding, horseradish peroxidase-conjugated anti-mouse F(ab′)2 fragments (1:5000 in PBS with 0.3% BSA; Abcam, Cambridge, UK). The membranes were reprobed with an antibody against α-tubulin (1.5000; Cell Signaling Technology) and HRP-conjugated anti-mouse F(ab′)2 to verify equal loading. Detection of protein bands was performed using ECL (Super Signal West Pico) reagent as recommended by the supplier (Pierce; Thermo Fisher Scientific, Henningsdorf, Germany). As a control, we used HELF Fi301 after 168 h p.i.

### 2.10. Co-Culturing of Caco-2 and HELF Fi301

Co-cultivation of Caco-2 with HELF Fi301 was at a ratio of 2:1 (in 10% FBS EMEM for 48 h, 1% FBS EMEM for 24 h). Caco-2 mono-(6 × 10^4^ per well) and co-cultures (4 × 10^4^ Caco-2, 2 × 10^4^ HELF Fi301) were seeded in 24-well plates. After 72 h the cells were treated with EGTA followed by infection with HCMV (MOI of 10) and overlaid with methyl cellulose containing 2.77, 4.01, or 5.51 nM Bortezomib. At the same time, freshly harvested, untreated Caco-2 cells (4 × 10^4^) were mixed with HCMV-infected (MOI of 1) HELF Fi301 (2 × 10^4^) in methyl cellulose. After incubation for seven days plaques were counted and the reduction calculated.

### 2.11. Apoptosis Assay

Flow cytometry analysis was used to evaluate cell apoptosis. Briefly, Caco-2 cells seeded in 24-well plates and cultured in medium with 2% FBS, were infected with HCMV (MOI: 10) or mock-infected. After 6.0 days of infection, the cells were harvested and the degree of apoptosis and necrosis was determined by staining with annexin V as well as propidium iodide and IE 1/2 using the FITC Annexin V Apoptosis Detection Kit with PI (BioLegend, San Diego, USA). After cytofluorimetric analyses data were imaged using the software FlowJo V 10.7.1 (BD bioscienes-EU, Eysins, Switzerland)).

### 2.12. Mitochondrial Membrane Potential Assay

Early stages of cell death induce mitochondrial disruption resulting in changes of membrane potential. In order to monitor this effect fluorescent imaging with JC-1 dye, a mitochondrial membrane indicator, was performed. JC-1 dye aggregation in the mitochondria is potential dependent, and leads to an emission shift from green (ʎ 527 nm) to red (ʎ 590 nm) (monomer to aggregates). We used the JC-1-Mitochondrial Membrane Potential Assay Kit (Cayman, Biomol, Hamburg, Germany).

Caco-2 cells grown in a 96-well microplate (1.5 × 10^4^ per well) were infected with HCMV (MOI 10) or mock-infected. At 6–6.5 days p.i. the cells were treated with 4.01 nM (EC_50_) or 5.51 nM (EC_75_) Bortezomib for 12 h and 24 h. Mock-infected or untreated HCMV-infected cells were used as controls. The assay was performed in duplicate according to the instructions of the supplier (Abcam, Cambridge, UK). The fluorescent ratio of infected to mock-infected Caco-2 was used for normalization.

### 2.13. Cell-Death Detection

In order to quantitative measurements of apoptosis the Cell Death Detection ELISAPlus (Roche, Mannheim, Germany) was used. This assay detect histone-associated DNA fragments, which determined the intranucleosomal degradation of DNA during apoptosis. The quantitative sandwich-ELISA used monoclonal antibodies directed against DNA and histones. Mock-infected or infected Caco-2 cells were treated with Bortezomib (4.01 nM or 5.51 nM) or left untreated (medium with 2% FBS) for 12 and 24 h. The cells were trypsinized, washed, and seeded (in duplicates) at 1 × 10^5^ cells/wells. The assay was performed according to manufacturer’s instructions. Absorption was measured at a wavelength of 405 nm (reference wavelength: 492 nm).

### 2.14. Necrosis Assay with PicoGreen

In order to quantify double-stranded DNA the Pico Green assay (Molecular Probes Europe BV, Leiden, Netherland) was used. With this assay dsDNA is detected by using an ultrasensitive fluorescent nucleic acid stain. Mock-infected or infected Caco-2 cells were treated with Bortezomib (4.01 nM or 5.51 nM) or left untreated (medium with 2% FBS) for 12 and 24 h. Supernatants were harvested and soluble dsDNA was analyzed in comparison to a Lambda-DNA standard. The fluorescence emission intensity was measured at 535 nm (excitation: 485 nm).

### 2.15. Cell Viability

The trypan blue dye exclusion test was used to determine the number of viable cells in cell suspension. The assay is based on the principle that viable cells possess intact membranes that exclude the dye, whereas dead cells do not. Equal volumes of cell suspensions and 0.4% trypan blue solution (Thermo Fisher Scientific, Waltham, MA, USA) were mixed. After an incubation period of approx. 3 min at RT the assay was visually examined to determine whether cells take up (nonviable cells) or exclude dye (viable cells). The assay was performed in triplicates.

### 2.16. Statistical Analysis

All experiments were performed in duplicate in 3–4 biological replicates. Data were expressed as mean ± standard deviation (SD). Differences were evaluated by Student’s *t*-test using the software GraphPad Prism^®^ V 8.4.3 (GraphPad Software Inc., La Jolla, CA, USA), *p*-values are denoted as follows: * *p* < 0.05, ** *p* < 0.01, *** *p* < 0.001, **** *p* < 0.0001.

## 3. Results

### 3.1. Susceptibility of Caco-2 Cells to HCMV

**Figure 1:**
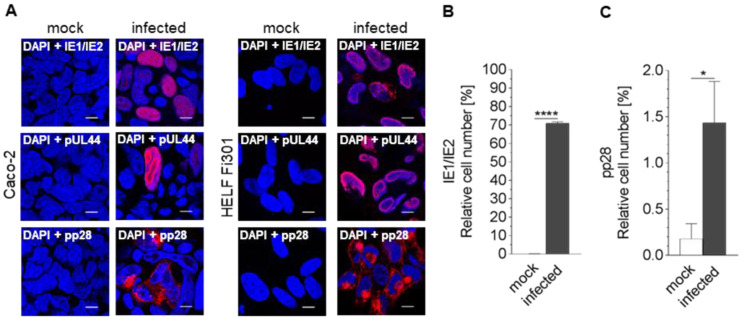
Qualitative and quantitative analysis of HCMV protein expression in Caco-2 cells. (**A**) Caco-2 cells were uninfected (mock) or infected with HCMV. After 7d p.i. the cells were subjected to immunofluorescence using antibodies against IE1/IE2 (immediate early), pUL44 (early) and pp28 (late). Mock-infected cells served as control. Size standard: 10 μm. As a control we used mock-infected and infected HELF Fi301. Mock-infected and HCMV infected Caco-2 at 7d p.i. were subjected to flow cytometry using antibodies against IE1/IE2 (**B**) or pp28 (**C**) in order to quantify percent of infection. Values represent mean ± SD from three independent experiments. **** *p* < 0.0001, * *p* < 0.05.

In order to demonstrate the ability of HCMV to infect the colon carcinoma cell line Caco-2 infected cells were analyzed by immunofluorescence. Analyses included immediate early (IE; IE1), early (E; pUL44), and late (L; pp28) protein expression. Cover slip cultures were infected for 7 d with a HCMV isolate (MOI 10) or mock-infected before fixation and immunofluorescence. Expression of IE1 as well as pUL44 and pp28 was observed in Caco-2 cells (Figure 1A), thus showing the permissivity of Caco-2 for HCMV. For quantification of the results, a flow cytometry analysis using IE1/IE2 and pp28 staining was established. The immediate early proteins IE1/IE2 were detected in infected cultures. Mock-infected cells served as controls. The analysis revealed that up to 72% of Caco-2 were infected with HCMV compared to mock-infected cells (Figure 1B). Furthermore, analysis with antibodies against the tegument protein pp28 confirmed the ability of Caco-2 to express all classes of HCMV genes (Figure 1C). Caco-2 cells therefore showed semi-permissivity to HCMV infection.

**Figure 2:**
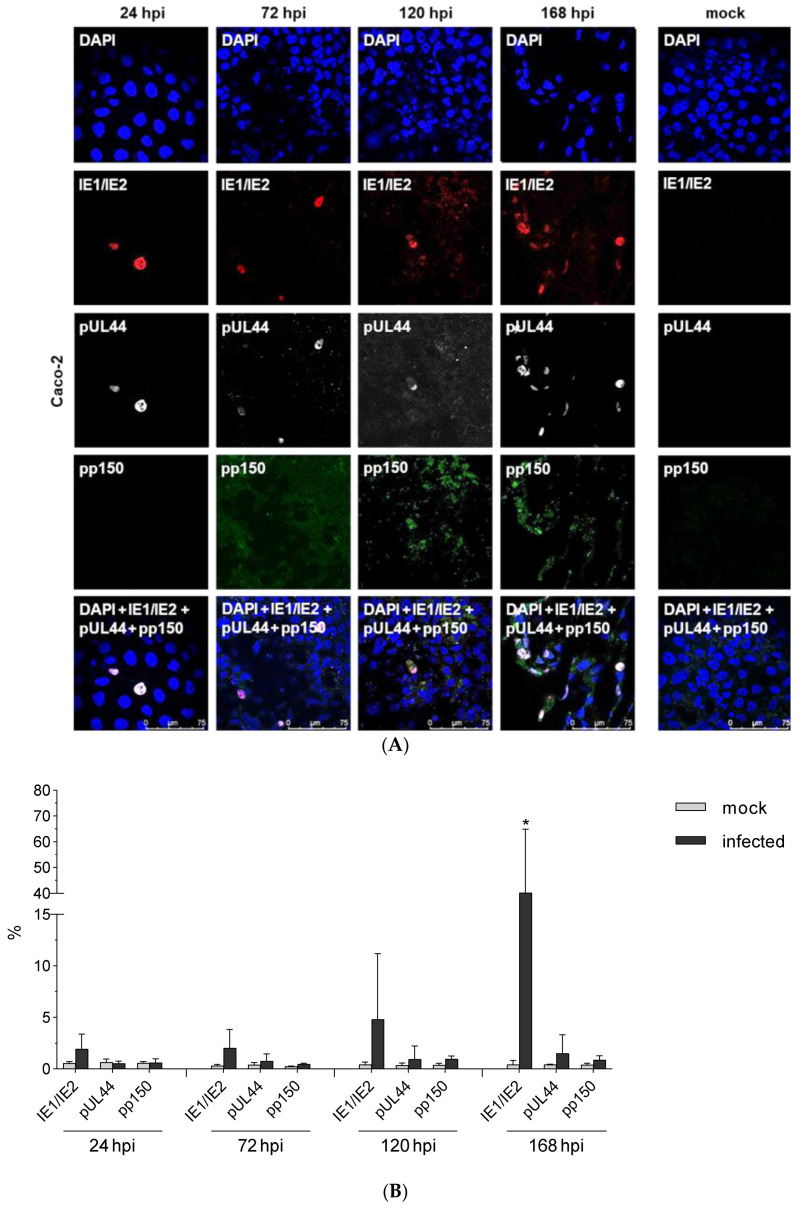
Qualitative and quantitative kinetic analysis of HCMV protein expression during the infectious cycle in Caco-2 cells. (**A**) Caco-2 cells were infected with HCMV-TB40/E-pp150EGFP (MOI 10) and after 1, 3, 5, and 7 d p.i. subjected to immunofluorescence. Staining was performed by using GFP auto-fluorescence (Green, pp150, Late antigen) and antibodies against IE1/2 (Red, DyLight 549, Immediate early antigen) and pUL44 (White, Alexa Fluor 647, Early antigen). Mock-infected cells (168 hpi) served as control.Scale bar: 75 µm (**B**) Mock-infected and HCMV infected Caco-2 were after 1, 3 5 and 7d p.i. subjected to flow cytometry using antibodies against IE1/IE2 or pUL44 as well as GFP autofluorescence in order to quantify the percent of infection. Values represent mean ± SD from three independent experiments. * *p* < 0.05.

To investigate the protein expression in the viral life cycle Caco-2 cells were infected with HCMV TB40/E-pp150EGFP and analyzed by immunofluorescence at 1d, 3d, 5d, and 7d p.i. Analysis included immediate early (IE; IE1/2; red), early (E; pUL44; ultraviolet) and late (L; pp150; GFP) protein expression. Mock-infected cells served as control. As anticipated, expression of IE1/2 and early proteins were detected during the whole time scale (Figure 2A), while pp150 expression was detected at day 5 p.i. (Figure 2B). The quantitative FACS analysis confirmed these data. Expression of IE 1/2 increased during infection from 1.56% at day 1 to 61.3% at day 7 and pUL44 increased from 0.49% at day 1 to 3.61% at day 7. While expression ofpp150 was delayed and started at day 5 p.i. and increased to 1.21% at day 7 p.i. These experiments demonstrated that HCMV replicates in Caco-2 cells and leads to approximately 61% infection of the cells.

### 3.2. Growth Kinetics

**Figure 3:**
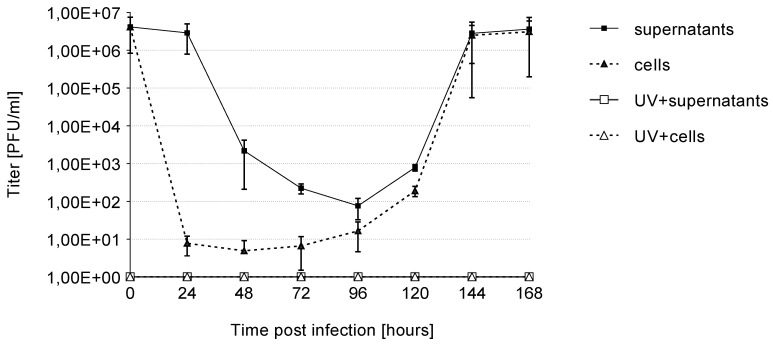
Growth kinetics of HCMV or UV irradiated HCMV infected Caco-2 cells. At each time point, cells and supernatants were harvestested and used for infection of HELF Fi301. As a control supernatants and cells from UV-iradiated virus were used. Progeny virus titers were determined by plaque reduction assay. Error bars reprent standard deviations (SD) from three independent experiments.

To determine whether HCMV is able to replicate in Caco-2 cells, we harvested cells and supernatants of HCMV or UV-irradiated HCMV (MOI 10) infected Caco-2 cells up to 168 h p.i. The supernatants and cells were used to infect HELF Fi301. Viral titers were determined by plaque reduction assay at indicated time points (Figure 3). Harvested supernatants and cells from infected Caco-2 were able to infect HELF Fi301, while supernatants and cells of UV-inactivated virus failed to replicate. At early time points viral yield decreases as expected (Figure 3). Viral replication initiated at 96 h p.i.. After 7 day p.i. viral yield increases to high viral titers. (Figure 3). In conclusion, it was demonstrated that HCMV infections in Caco-2 release infected viruses that could infect HELF Fi301.

### 3.3. Expression Kinetic of IE1/2

**Figure 4:**
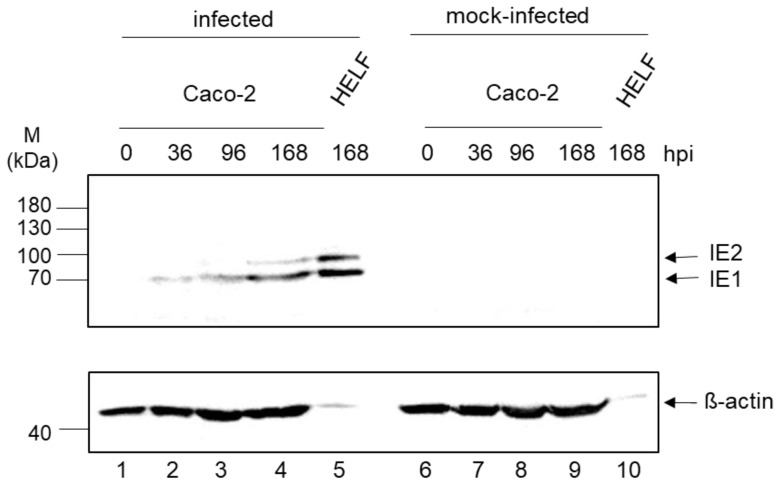
Expression kinetic of IE1/IE2 in Caco-2 cells. HCMV-infected (lanes 1–4) and mock-infected Caco-2 cells (lanes 6–9) were harvested at indicated time after infection (0, 36, 96, 168 h p.i.). HCMV infected HELF Fi301 (lane 5) and mock-infected (lane 10) were harvested at 168 h p.i. Extracts were separated by 10% SDS-PAGE and transferred to nitrocellulose. The immunoblot was reacted with antibodies against IE1/2. Actin served as a loading control. Markers (kDa) are indicated on the left, the positions of IE proteins on the right. The experiment was performed three independent times.

To investigate the protein expression of IE1 and IE2 western blot analysis was carried out. HCMV-infected Caco-2 cells were harvested at 0, 36, 96, and 168 h p.i, infected HELF Fi301 at 168 h p.i. and subjected to immunoblot analysis using antibodies against immediate-early proteins IE1 and IE2. Equal amount of proteins in all Caco-2 samples were confirmed by using β-actin as loading control. Expression of IE1 72 kDa and IE2 82 kDa was detected and increased during ongoing infection (Figure 4).

### 3.4. Genome Quantification

To further investigate the permissivity of Caco-2 cells the amount of HCMV genome was quantified by real-time PCR. Mock-infected and infected cells were harvested at 36, 96, and 168 h p.i. DNA was extracted and used for quantification of pp150 DNA copy numbers. During infection pp150 copy numbers increased (Table 1). The significance of the results was provided by biostatistics analysis by comparison with copy numbers of TB40/E BAC4 (Table 1). These results showed that Caco-2 cells are susceptible for HCMV infection.

### 3.5. Antiviral Effect of Bortezomib on HCMV Infected Caco-2 Cells and Cytotoxicity

In order to prove the antiviral activity of bortezomib, plaque reduction assay were performed. The results of the plaque reduction assays provided evidence for anti-HCMV activity. Cells treated with various concentrations of bortezomib were dose dependently protected against infection.

**Figure 5:**
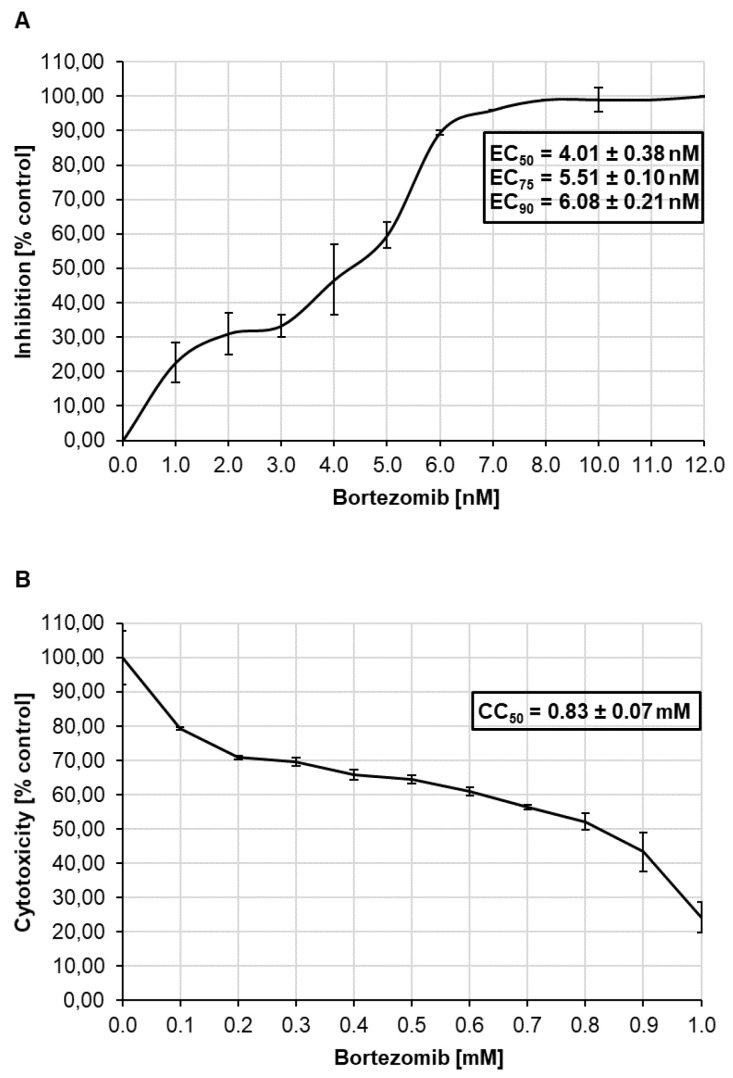
Bortezomib inhibits HCMV infection of Caco-2 cells and its cytotoxicity. (**A**) Caco-2 cells were infected with the clinical isolate (MOI 10) in the presence of increasing concentrations of Bortezomib. At 7 d p.i. plaque reduction assay was performed. Plaque reduction is indicated as inhibition as a percentage of PFU obtained in the absence of Bortezomib. The mean 50% (EC_50_), 75% (EC_75_), and 90% (EC_90_) effective concentrations range from 4.01 to 6.08 nM. Results were obtained from three independent experiments. Error bars represents the standard deviations. (**B**) Various concentrations of Bortezomib (mM) were added to Caco-2 cells. At 24 h, XTT cell proliferation assay was perfomed. Values are represented as percentage of untreated control cells. Data are mean values from three independent experiments. Error bars respresent the standard deviations.

Theeffective concentrations (EC) of Bortezomib against HCMV were for mean EC_50_ value 4.01 ± 0.38 nM, the mean EC_75_ value 5.51 ± 0.10 nM and the mean EC_90_ value 6.08 ± 0.21 nM (Figure 5A).

The cytotoxicity of Bortezomib was investigated to exclude that unspecific effects could affect its anti-HCMV activity. We exposed Caco-2 cells to several doses of Bortezomib. To determine the cytotoxicity of Bortezomib an XTT cell proliferation assay was performed. The mean 50% cytotoxic concentration (CC_50_) was 0.8.3 ± 0.07 mM after 24 h of treatment (Figure 5B). This results demonstrated that bortezomib exhibits no cytotoxicity at concentration that are effective against HCMV.

### 3.6. Effects of HCMV and Bortezomib on Apoptosis in Caco-2 Cells

**Figure 6:**
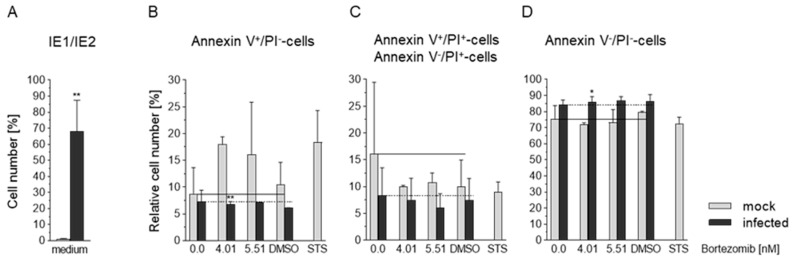
Effects of HCMV or Bortezomib on apoptosis in Caco-2 cells using flow cytometry. Mock-infected and HCMV isolate infected Caco-2 cells were treated with medium or Bortezomib, the solvent DMSO or staurosporin (STS) for 4 h. Half of the probe was used for detection of IE1/2 (**A**). The stages of apoptosis are shown (**B**) early stage (percentage of Annexin V^+^/PI^−^cells), (**C**) late stage (Annexin V^+^/PI^+^) and Annexin V^−^/PI^+−^ cells), and (**D**) viable cells (Annexin V^−^/PI^−^ cells). In (**B**), (**C**), and (**D**), the solid line indicate as references the position of the untreated mock-infected and the broken line those of the untreated infected cells. Values represent mean ± SD from two independent experiments. ** *p* < 0.01, * *p* < 0.05.

**Figure 7:**
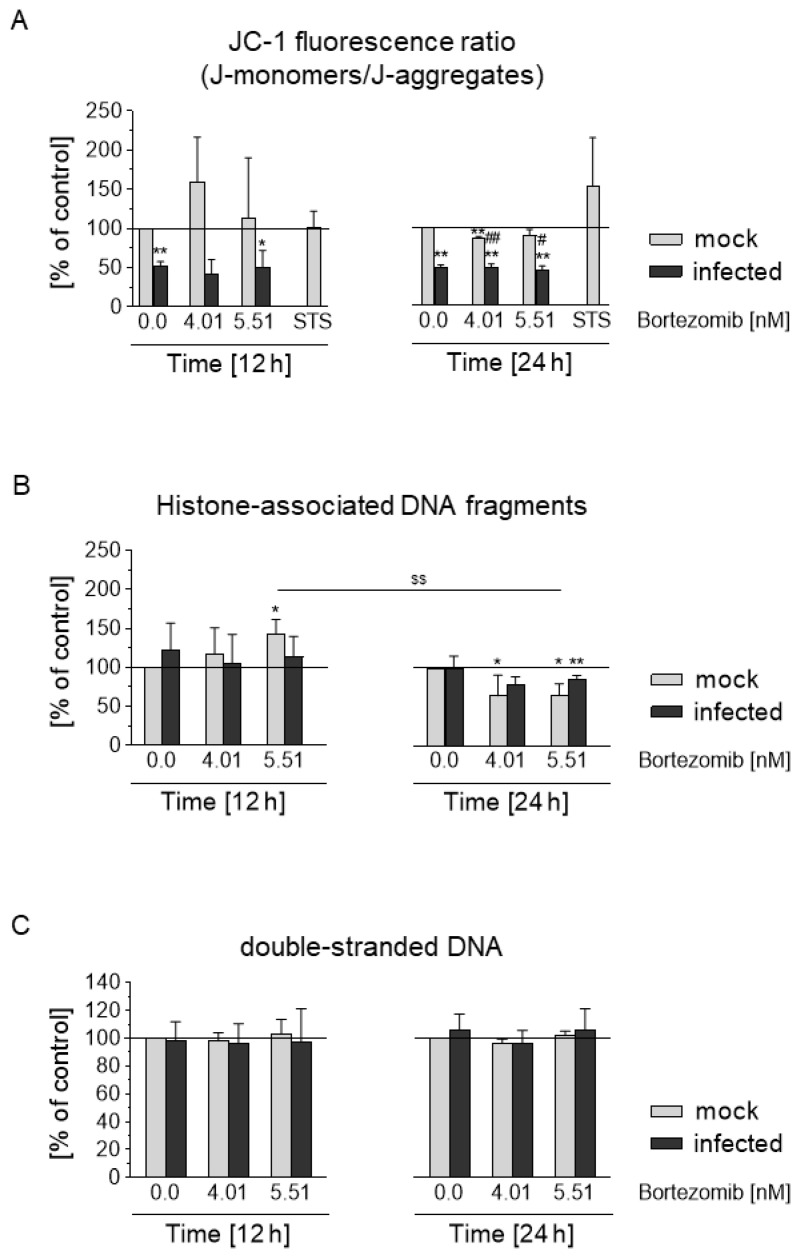
Effects of HCMV or Bortezomib on early stages of cell death in Caco-2 cells. (**A**) Mock-infected and HCMV isolate infected Caco-2 cells were treated with Bortezomib (4.01 nM (EC_50_); 5.51 nM (EC_75_) or left untreated (0.00 nM). 12 and 24 h after treatment JC-1 Mitochondrial Membrane Potential assay was performed. The JC-1 fluorescence ratio (%) of infected and proteasome inhibitor-treated mock-infected and infected Caco-2 was normalized against that of the mock-infected control (defined as 100%, solid line). (**B**) The infection as well as the treatment of the cells was identical to (**A**). The values of the absorption of the Cell Death Detection ELISAPlus were shown as percentage of the control. (**C**) From the cell culture supernatants of the above described cell treatments, dsDNA concentration was determined by PicoGreen^®^ assay. Values represent mean ± SD from three independent experiments. *^,^** Untreated infected Caco-2, bortezomib-treated mock-infected and infected Caco-2 compared with mock-infected Caco-2. ^#,##^ Comparison of bortezomib-treated infected with bortezomib-treated mock-infected Caco-2. ^$$^ 24-h bortezomib treatment of mock-infected Caco-2 was compared to the corresponding 12-h treatment. *^,#^ *p* < 0.05 **^,##,$$^ *p* < 0.01.

To prove whether HCMV infection and/or Bortezomib have a direct effect on apoptosis Annexin V binding, propidium iodide (PI) uptake, and presence of virus (IE staining, Figure 6A) was analyzed. Early apoptosis was defined as Annexin V^+^/PI^−^ cells (Figure 6B), late apoptosis as Annexin V^+^/PI^+^ and Annexin V^−^/PI^+-^ cells (Figure 7C), and viable cells as Annexin V^−^/PI^−^ cells (Figure 6D). Mock-infected and infected Caco-2 (MOI 10) were treated with increasing amounts of Bortezomib at 6 days p.i. and the analysis was subsequently performed at 4 h post treatment. Medium, DMSO (the solvent of the proteasome inhibitor), and staurosporin (STS) served as controls. In infected cells, a decrease in the apoptotic population (Annexin V^+^/PI^−^) was observed. Identical effects were detected in the late stage of apoptosis (Annexin V^+^/PI^+^ and Annexin V^−^/PI^+^) (Figure 6B). Treatment of cells with Bortezomib has an pro-apoptotic effect which is blocked by HCMV (Figure 6B,C). Cell survival rates (Annexin V^−^/PI^−^) reflect the results described above (Figure 6D). Overall, it was shown that (i) HCMV has a significant anti-apoptotic effect on Caco-2, (ii) Bortezomib was not able to overcome this effect despite effectively inducing apoptosis in uninfected cells.

### 3.7. HCMV Prevents Depolarization of the Mitochondrial Membrane and Blocks the Apoptotic Effect of Bortezomib

To investigate the influence of HCMV and Bortezomib on very early stages of apoptosis, the JC-1 assay was used (Figure 7A). The ratio of staining of JC-1 dye is dependent on mitochondrial membrane potential and changes indicate activation of the intrinsic pathway of apoptosis resulting in loss of membrane potential. The infection and cultivation of Caco-2 was carried out as described under 2.3. Subsequently, the cells were treated with Bortezomib (4.01 nM [EC_50_], 5.51 nM [EC_75_]) for 12 h and 24 h or left untreated. The JC-1 fluorescence ratio (%) of infected and proteasome inhibitor-treated mock-infected and infected Caco-2 was normalized against that of the mock-infected control. At both time points HCMV-infected cells induced a significant decrease in the green/red fluorescence intensity ratio compared to the mock-infected cells (Figure 7A, w/o). Bortezomib showed the same trend in the infected Caco-2, and in addition after 24 h a decrease in mock-infected cells. Moreover, after 12 h treatment of mock-infected cells with Bortezomib an increase was observed (Figure 7A). Interestingly, after 24 h treated mock-infected cells showed a significantly reduced effect. Staurisporin was used as control. These results demonstrated that HCMV infection prevents the depolarisation of the mitochondrial transmembrane regardless of the concentration of Bortezomib.

To further measure the extent of apoptosis, histone-associated DNA fragments of the cytoplasmic fraction of cell lysates were detected by Cell Death Detection ELISAPlus (Figure 7B). In addition, cell culture supernatants were used to assess necrosis of Caco-2 by determination of dsDNA using the PicoGreen^®^ assay (Figure 7C).

The analysis of histone-associated DNA revealed that HCMV leads to a reduced effect on Caco-2 after 24 h (Figure 7B). Treatment of mock-infected cells with inhibitor leads to an increase of histone-associated DNA after 12 h, while after 24 h a decrease was observed. After 24 h, treatment with Bortezomib infected cells showed a decrease of histone-associated DNA (Figure 7B). Further analysis of dsDNA demonstrated that the effects of HCMV and Bortezomib are not due to necrosis (Figure 7C). In conclusion, these studies showed that in the cancer cells HCMV had an inhibitory effect on very early events of the apoptosis cascade. In the mock-infected Caco-2, Bortezomib initially showed a slight pro-apoptotic effect which over time became anti-apoptotic. At later stages of apoptosis both HCMV and Bortezomib show a similar trend.

### 3.8. HCMV Infection of Caco-2 by Co-Culture with Infected HELF Fi301

HCMV in vivo is efficiently spread via cell-to-cell infection. Tumor cells are often found in a matrix of HCMV-permissive fibroblasts and other cells. In order to investigate the infection rate of the co-culture (Caco-2 and HELF Fi301)) with those of the Caco-2 mono-culture, all cells were infected (MOI 10). Furthermore, the infection of Caco-2 was carried out with co-culturing HCMV-infected HELF Fi301 (mixed in the ratio 2:1). The fibroblasts were used after obtaining a 100% CPE. After 7d p.i. plaque assays were performed. For both co-cultures (infected co-culture, Caco-2 + infected HELF Fi301) significantly higher total infection rates were detectable compared to those of the infected mono-culture (Figure S1A). In conclusion, the results demonstrate that the co-culture of Caco-2 with HCMV-infected HELF Fi301 is an efficient and effective method of infection. In addition, depending on the number of cells as well as on the HCMV titer, either a cytopathic effect (CPE) or a proliferation increase with a high infection rate could be induced in the Caco-2 cell line (Figure S1B).

### 3.9. Effects of Co-Culture on Apoptosis

**Figure 8:**
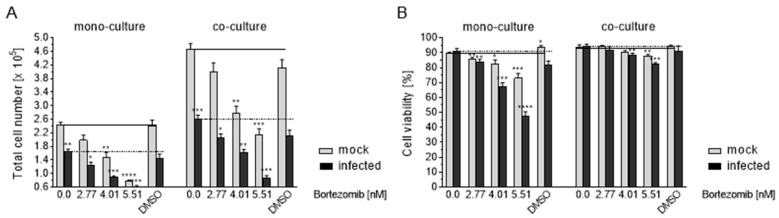
Proliferation and viability effects of Caco 2 mono- and co-cultures. Mock-infected and infected Caco-2 cells were treated with Bortezomib, the solvent DMSO or left untreated. Seven days after treatment, proliferation and viability were analyzed using the trypan blue exclusion assay. (**A**) Total number of cells (× 10^5^) of untreated, Bortezomib treated and the solvent treated cultures. (**B**) Cell viability (%). In the graph, the solid line indicates the level of the values of the mock-infected control of the Caco-2 mono- and co-cultures and the dashed line those of the infected cultures. *, ** Infected vs. mock-infected Caco-2 and bortezomib-treated mock-infected and infected Caco-2 vs. appropriately infected Caco-2. Values represent mean ± SD from three independent experiments. * *p* < 0.05, ** *p* < 0.01, *** *p* < 0.001, **** *p* < 0.0001.

The trypan blue exclusion test was used to examine the proliferation and viability of mock-infected and infected Caco-2 mono- and co-cultures. The cultures were treated for 7 days with increasing amounts of Bortezomib. Untreated cells (w/o) and treatment with the solvent DMSO served as controls. HCMV infection resulted in a significantly reduced cell proliferation in the mono-culture compared to the mock-infected control. In comparison to the mono-culture, the mock-infected co-culture showed a much higher effect, whereas the infected co-culture had a higher significant effect (Figure 8A). Bortezomib treatment led to a reduced cell proliferation in the mono- as well as the co-culture, depending on the concentration (Figure 8A,). Even more, the effect of bortezomib was significantly reduced by HCMV infection (Figure 8A). Untreated mock-infected as well as infected co-culture showed a similar viability (Figure 8B, left). For the proteasome inhibitor Bortezomib, apoptotic effects were found in the mono-culture depending on the concentration (Figure 8B, left). In co-cultures, this effect in cells treated with Bortezomib was significant but less pronounced (Figure 8B, right).

## 4. Discussion

In order to demonstrate the ability of clinical isolates, as well as a low passaged GFP-tagged pp150 HCMV, to infect colon carcinoma cell lines, detection of immediate early, early, and late proteins were performed in infected cells. By immunofluorescence and flow cytometry all classes of viral proteins were observed. Growth kinetics demonstrated that virus released from Caco-2 cells is able to infect HELF. The phenomenon of viral replication enhancement by high initial titers is well known in cytomegalovirus research, particularly with IE1 deficient viruses [39]. This implies that the principle barrier to infection in this cell type is immediately post-infection. In addition, it was shown by plaque reduction assay that infected Caco-2 cells were able to infect neighboring cells by cell-to-cell spread. This is in contrast to the observation of Jarvis et al. [40] where virus (MOI 25; strain Towne) was released from the cells but cell-to-cell spread was not observed. Interestingly, by using the laboratory HCMV strain AD169 Esclatine et al. [41] were able to infect Caco-2 cells. This group observed 40% IE positive cells and 5% E- and L-positive cells. In contrast to laboratory strains, our HCMV clinical isolate as well as TB40/E-pp150EGFP are able to infect a wider range of cell types due to retention of the gH/gL/UL128/UL130/UL131 pentamer. The use of alternative receptors explains higher infection rates of approximately 70%. Furthermore, it has been demonstrated that infection rates also affected by access of HCMV to the cell surface receptor [42]. The presence of the pentamer could therefore enable HCMV to bind efficiently to the cell surface receptor. Laboratory strains lose their ability to infect many cell types because of the passage of viruses in fibroblasts leading to an accumulation of mutations [2]. Similar to our results Ryckman et al. [43] demonstrated that they were able to infect more cells (90% of epithelial ARPE-19) by using a low passage HCMV isolate. In summary, we could show that Caco-2 cells were susceptible to HCMV low passaged viruses. Furthermore, we demonstrated that infected Caco-2 cell are able to infect HELF Fi301 thus confirming that Caco-2 cells are semi-permissive for HCMV. Increasing protein expression as well as virus genome in infections underline the semi-permissiveness of Caco-2 cells.

Many viruses encode proteins that inhibit apoptosis, one of the main function of the innate defence mechanism against viral infection. It is known that HCMV encodes several cell death inhibitors (e.g., vMIA, vICA) that overcome mitochondrion-mediated apoptosis [24,25,26]. vMIA inactivates Bax by direct binding causing changes of Bax as well as mitochondrial translocation [24,43]. In addition, it has been reported that vMIA has another anti-apoptotic effect (Bax-independent), it leads to mitochondrial hyperpolarization [24]. In the present study, we provide evidence that in the colon-carcinoma cell line HCMV infection has a significant inhibitory effect on apoptosis. Our results showed that in the cancer cells HCMV stabilizes the mitochondria membrane thus preventing the depolarization of the membrane. These findings are in line with the known function of vMIA [24,43,44].

Furthermore, herpesviruses such as EBV [45], HSV-1 [46], and HCMV [47] have evolved mechanisms to inhibit necroptosis, thus preventing host-antiviral defense. Necroptosis is an alternative programmed cell death induced by signaling complexes containing receptor-interacting protein kinase-1 (RIPK1) and –kinase-3 (RIPK3). This form of cell death is thought to be a host defense to eliminate pathogen-infected cells. HCMV has developed a strategy distinct from RHIM signaling. HCMV blocks RIP3-dependent necroptosis by suppression with an IE-1 regulated protein [47]. However, the complete mechanism is still unknown. In the present study, Bortezomib showed a pro-apoptotic (necroptosis) effect in colon carcinoma cells.

Overall, an active proteasome is a prerequisite for multiple events in HCMV life cycle. In this regard, Bortezomib is an attractive target for therapeutic interventions. In addition, Bortezomib is widely used for treatment of patients with multiple myeloma. A Bortezomib-containing regiment could be either as a single drug or in combination. However, increasing numbers of studies have showed that multiple myeloma patients treated with Bortezomib-based regimens are at higher risk of developing a symptomatic CMV reactivation after autologous stem cell transplantation [48,49,50]. Li et al. [51] provided evidence that Bortezomib reduce the number of T lymphocytes resulting in susceptibility to HCMV infection. Together with our findings, this suggests that treatment with Bortezomib in HCMV positive patients could lead to a failure of therapy. Furthermore, our in vitro data in Caco-2 cells points to the unsuitability of Bortezomib as an anti-cancer drug against colon tumors in the presence of HCMV.

In order to model the in vivo situation where tumor cells are surrounded by cancer-associated fibroblasts (CAFs), we used co-cultivation studies with HELF and Caco-2 [52]. CAFs facilitate the growth, proliferation, and cell invasion of tumors [53,54]. CAFs are a subpopulation of cells of the tumor microenvironment and important for encouraging tumor progression [55]. Interestingly, we could show that co-cultivation of HCMV infected HELF Fi301 with Caco-2 cells leads to infection of Caco-2 cells. Even more, supernatants from infected Caco-2 cells infect HELF cells. Thus leading to the hypothesis that HCMV can invade tumor cells and may induce tumor progression by preventing apoptosis and necroptosis.

## Figures and Tables

**Table 1:** HCMV pp150-DNA (copies per 500 ng) normalized to TB40/E BAC4.

Template	Time Post Infection (h)		
	36	96	168
Inf. Caco-2	1.724 × 10^3^	3.273 × 10^3^	4.305 × 10^3^

## Data Availability

The data presented in this study are available on request from the corresponding author. The data are not publicity available due to restricted access to the server of the Charité-Universitätsmedizin Berlin.

## Supplementary Materials

The following are available online at https://www.mdpi.com/1999-4915/13/1/83/s1, Figure S1: Effects of co-culture and co-infection on the HCMV infection of the Caco-2 cells.

## References

[1] Britt W.J., Alford C.A., Fields B.N., Knipe D.M., Howley P.M. (1996). Cytomegalovirus. Fields Virology.

[2] Mocarski E.S., Shenk T., Griffiths P.D., Pass R.F., Knipe D.M., Knipe D.M., Howley P.M. (2013). Cytomegalovirus. Fields Virology.

[3] Adams M.J., Carstens E.B. (2012). Ratification vote on taxonomic proposals to the International Committee on Taxonomy of Viruses. Arch. Virol..

[4] Cobbs C.S., Harkins L., Samanta M., Gillespie G.Y., Bharara S., King P.H., Nabors L.B., Cobbs C.G., Britt W.J. (2002). Human cytomegalovirus infection and expression in human malignant glioma. Cancer Res..

[5] Harkins L., Volk A.L., Samanta I., Mikolaenko I., Britt W.J., Bland I., Cobbs S. (2002). Specific localisation of human cytomegalovirus nucleic acids and proteins in human colorectal cancer. Lancet.

[6] Mitchell D.A., Xie W., Schmittling R., Learn C., Friedman A., McLendon R.E., Sampson J.H. (2008). Sensitive detection of human cytomegalovirus in tumors and peripheral blood of patients diagnosed with glioblastoma. Neuro. Oncol..

[7] Samanta M., Harkins L., Klem K., Britt W.J., Cobbs C.S. (2003). High prevalence of human cytomegalovirus in prostatic intraepithelial neoplasia and prostatic carcinoma. J. Urol..

[8] Taher C., Taher C., Frisk G., Fuentes S., Religa P., Costa H., Assinger A., Vetvik K.K., Bukholm I.R., Yaiw K.C. (2014). High prevalence of human cytomegalovirus in brain metastases of patients with primary breast and colorectal cancers. Transl. Oncol..

[9] Chen H.P., Jiang J.K., Chen C.Y., Chou T.Y., Chen Y.T., Lin S.F. (2012). Human cytomegalovirus preferentially infects the neoplastic epithelium of colorectal cancer: A quantitative and histological analysis. J. Clin. Virol..

[10] Faivre J., Bouvier A., Bonithon-Kopp C. (2002). Epidemiology and screening of colorectral cancer. Best Pract. Res. Clin. Gastroenterol..

[11] Chen H.P., Jiang J.K., Lai P.Y., Chen C.Y., Chou T.Y., Chen Y.C., Chang Y.T., Lin S.F., Chan C.H., Yang C.Y. (2014). Tumoral presence of human cytomegalovirus is associated with shorter disease-free survival in elderly patients with colorectal cancer and higher levels of intratumoral interleukin-17. Clin. Microbiol. Infect..

[12] Chen H.P., Jiang J.K., Chen C.Y., Yang C.Y., Chen Y.C., Lin C.H., Chou T.Y., Cho W.L., Chan Y.J. (2016). Identification of human cytomegalovirus in tumour tissues of colorectal cancer and its association with the outcome of non-elderly patients. J. Gen. Virol..

[13] Chen H.P., Jiang J.K., Lai P.Y., Teo W.H., Yang C.Y., Chou T.Y., Lin C.H., Chan Y.J. (2016). Serological and viraemic status of human cytomegalovirus infection in patients with colorectal cancer is not correlated with viral replication and transcription in tumours. J. Gen. Virol..

[14] Cinatl J., Vogel J.U., Kotchetkov R., Doerr H. (2004). Oncomodulatory signals by regulatory proteins encoded by human cytomegalovirus: A novel role for viral infection in tumor progression. FEMS Microbiol. Rev..

[15] Salvant B.S., Fortunato E.A., Spector D.H. (1998). Cell cycle dysregulation by human cytomegalovirus: Influence of the cell cycle phase at the time of infection and effects on cyclin transcription. J. Virol..

[16] Michaelis M., Kotchetkov R., Vogel J.-U., Doerr H.W., Cinatl J. (2004). Cytomegalovirus infection blocks apoptosis in cancer cells. Cell. Mol. Life Sci..

[17] Karki K., Haradhchandra S., Sage S. (2018). Bortezomib targets Sp transcription factors in cancer cells. Mol. Pharmacol..

[18] Hong S.W., Kim S.M., Jin D.H., Shin J.S., Yoon D.H., Kim K.P., Lee J.L., Heo D.S., Lee J.S., Kim T.W. (2012). Bortezomib induces G2-M arrest in human colon cancer cells through ROS-inducible phosphorylation of ATM-CHK1. Int. J. Oncol..

[19] Zimmermann C., Büscher N., Krauter S., Krämer N., Sehn E., Tenzer S., Plachter B. (2018). The abundant tegument protein pUL25 of human cytomegalovirus prevents proteasomal degradation of pUL26 and supports its suppression of ISGylation. J. Virol..

[20] Biolatti M., Dell’Oste V., Pautasso S., Gugliesi F., von Einem J., Krapp C., Jakobsen M.-R., Borgogna C., Gargilio M., De Andrea M. (2018). Human cytomegalovirus tegument protein pp65 (pUL83) dampens type I interferon production by inactivating the DNA sensor cGAS without affecting STING. J. Virol..

[21] Kim J.E., Kim Y.E., Stinski M.F., Ahn J.H., Song Y.J. (2017). Human cytomegalovirus IE2 86 kDa protein induces STING degradation and inhibits cGAMP-mediated IFN-β induction. Front. Microbiol..

[22] Lee S.H., Kalejta R.F., Kerry J., Semmes O.J., O’Connor C.M., Khan Z., Garcia B.A., Shenk T., Murphy E. (2012). BclAF1 restriction factor is neutralized by proteasomal degradation and microRNA repression during human cytomegalovirus infection. Proc. Natl. Acad. Sci. USA.

[23] Fennell D.A., Chacko A., Mutti L. (2008). BCL-2 family regulation by the 20S proteasome inhibitor bortezomib. Oncogene.

[24] Arnoult D., Bartle L.M., Skaletskaya A., Poncet D., Zamzami N., Park P.U., Sharpe J., Youle R.J., Goldmacher V.S. (2004). Cytomegalovirus cell death suppressor vMIA blocks Bax- but not Bak-mediated apoptosis by binding and sequestering Bax at mitochondria. Proc. Natl. Acad. Sci. USA.

[25] Brune W., Andoniou C.E. (2017). Die another day: Inhibition of cell death pathways by cytomegalovirus. Viruses.

[26] Chaudhry M.Z., Casalegno-Garduno R., Sitnik K.M., Kasmapour B., Pulm A.K., Brizic I., Eiz-Vesper B., Moosmann A., Jonjic S., Mocarski E.S. (2020). Cytomegalovirus inhibition of extrinsic apoptosis determines fitness and resistance to cytotoxic CD8 T cells. Proc. Natl. Acad. Sci. USA.

[27] Terhune S., Torigoi E., Moorman N., Silva M., Qian Z., Shenk T., Yu D. (2007). Human cytomegalovirus UL38 protein blocks apoptosis. J. Virol..

[28] Voutsadakis I.A. (2007). Pathogenesis of colorectal carcinoma and therapeutic implications: The roles of the ubiquitin-proteasome system and Cox-2. J. Cell. Mol. Med..

[29] Boccadoro M., Morgan G., Cavenagh J. (2005). Preclinical evaluation of the proteasome inhibitor bortezomib in cancer therapy. Cancer Cell Int..

[30] Levin A., Minis A., Lalazar G., Rodriguez J., Steller H. (2018). PSMD5 inactivation promotes 26S proteasome assembly during colorectal tumor progression. Cancer Res..

[31] Bonvini P., Zorzi E., Basso G., Rosolen A. (2007). Bortezomib-mediated 26S proteasome inhibition causes cell-cycle arrest and induces apoptosis in CD-30+ anaplastic large cell lymphomas. Leukemia.

[32] Roy S.S., Kirma N.B., Santhamma B., Tekmal R.R., Agyin J.K. (2014). Effects of a novel proteasome inhibitor BU-32 on multiple myeloma cells. Cancer Chemother. Pharmacol..

[33] Adams J. (2004). The development of proteasome inhibitors as anticancer drugs. Cancer Cell.

[34] Sampaio K.L., Cavignac Y., Stierhof Y.D., Sinzger C. (2005). Human cytomegalovirus labeled with green fluorescent protein for live analysis of intracellular Particle Movements. J. Virol..

[35] Smuda C., Bogner E., Radsak K. (1997). The human cytomegalovirus glycoprotein B gene (ORF UL55) is expressed early in the infectious cycle. J. Gen. Virol..

[36] Bhat M., Toledo-Velasquez D., Wang L., Malanga C.J., Ma J.K., Rojanasakul Y. (1993). Regulation of tight junction permeability by calcium mediators and cell cytoskeleton in rabbit tracheal epithelium. Pharm. Res..

[37] Deli M.A. (2008). Potential use of tight junction modulators to reversibly open membranous barriers and improve drug delivery. Biochim. Biohys. Acta.

[38] Turk S.R., Shipman C., Nassiri R., Genzlinger G., Krawczyk S.H., Townsend L.B., Drach J.C. (1987). Pyrrolo[2,3-d]pyrimidine nucleosides as inhibitors of human cytomegalovirus. Antimicrob. Agents Chemother..

[39] Hofmann H., Sindre H., Stamminger T. (2002). Functional interaction between the pp71 protein of human cytomegalovirus and the PML-interacting protein human Daxx. J. Virol..

[40] Jarvis M.A., Wang C., Meyers H.L., Smith P.P., Corless C.L., Henderson G.J., Vieira J., Britt W.J., Nelson J.A. (1999). Human cytomegalovirus infection of Caco-2 cells occurs at the basolateral membrane and is differentiation state dependent. J. Virol..

[41] Esclatine A., Lemullois M., Servin A.L., Quero A.-M., Geniteau-Legendre M. (2000). Human cytomegalovirus infects Caco-2 intestinal epithelial cells basolaterally regardless of the differentiation state. J. Virol..

[42] Ryckman B.J., Jarvis M.A., Drummond D.D., Nelson J.A., Johnson D.C. (2006). Human cytomegalovirus entry into epithelial and endothelial cells depends on genes UL128 to UL150 and occurs by endocytosis and low-pH fusion. J. Virol..

[43] Liu X., Li Y., Peng S., Yu X., Li W., Shi F., Luo X., Tang M., Tan Z., Bode A.M. (2018). Epstein-Barr virus encoded latent membrane protein 1 suppresses necroptosis through targeting RIPK1/3 ubiquitination. Cell Death Dis..

[44] Ma J., Endlich F., Bermejo G.A., Norris K.L., Youle R.J., Tjandra N. (2012). Structural mechanism of Bax inhibition by cytomegalovirus protein vMIA. Proc. Natl. Acad. Sci. USA.

[45] Hong C.T., Chau K.-Y., Schapira H.V. (2016). The cytomegalovirus protein pUL37x1 targets mitochondria to mediate neuroprotection. Sci. Rep..

[46] Guo H., Omoto S., Harris P.A., Finger J.N., Bertin J., Gough P.J., Kaiser W.J., Mocarski E.S. (2015). Herpes simplex virus suppresses necroptosis in human cells. Cell Host Microbe.

[47] Omoto S., Guo H., Talekar G.R., Roback L., Kaiser W.J., Mocarski E.S. (2015). Suppression of RIP3-dependent necroptosis by human cytomegalovirus. J. Biol. Chem..

[48] Marchesi F., Mengarelli A., Giannotti F., Tendas A., Anacierico B., Porrini R., Picardi A., Cerchiara E., Dentamaro T., Chierichini A. (2014). High incidence of post-transplant cytomegalovirus reactivations in myeloma patients undergoing autologous stem cell transplantation after treatment with bortezomib-based regimens: A survey from the Rome transplant network. Transpl. Infect. Dis..

[49] Kaya A.H., Tekgunduz E., Akpinar S., Batgi H., Bekdemir F., Kayikci O., Namdarogl S., Ulu B.U., Dal M.S., Cakar M.K. (2017). Is cytomegalovirus surveillance necessary for patients with low reactivation risk in an autologous hematopoietic cell transplantation setting?. Transpl. Proc..

[50] Sharpley F.A., De-Silva D., Mahmood S., Sachchithanantham S., Ramsay I., Mingo A.G., Worthington S., Hughes D., Mehta A., Kyriakou C. (2020). Cytomegalovirus reactivation after bortezomib treatment for multiple myeloma and light chain amyloidosis. Eur. J. Haematol..

[51] Li J., Li Y., Huang B., Zheng D., Chen M., Zhou Z. (2015). Drug-induced modulation of T lymphocytes as a potential mechanism of susceptibility to infections in patients with multiple myeloma during bortezomib therapy. Cell Biochem. Biophys..

[52] Neuzillet C., Tijeras-Rabbaland A., Ragulan C., Cros J., Patil Y., Martinet M., Erkan M., Kleeff J., Wilson J., Apte M. (2019). Inter- and intra-tumoural heterogeneity in cancer-associated fibroblasts of human pancreatic ductal adenocarcinoma. J. Pathol..

[53] Hwang R.F., Moore T., Arumugam T., Ramachandran V., Amos K.D., Rivera A., Ji B., Evans D.B., Logsdon C.D. (2008). Cancer-associated stromal fibroblasts promote pancreatic tumor progression. Cancer Res..

[54] Cavaco A.C.M., Rezaei M., Caliandro M.F., Lima A.M., Stehling M., Dhayat A., Haier J., Brakebusch C., Eble J.A. (2018). The interaction between laminin-332 and alpha3beta1 integrin determines differentiation and maintenance of CAFs, and supports invasion of pancreatic duct adenocarcinoma cells. Cancers.

[55] Madar S., Goldstein I., Rotter V. (2013). ‘Cancer associated fibroblasts’—More than meets the eye. Trends Mol. Med..

