# Supplementary material for: HCMV-Mediated Interference of Bortezomib-Induced Apoptosis in Colon Carcinoma Cell Line Caco-2
Source: Viruses. 2021 Jan 9;13(1):83. doi: 10.3390/v13010083 (PMC7827311; doi:10.3390/v13010083)
Supplement: Supplementary file 1 [file viruses-13-00083-s001.pdf]

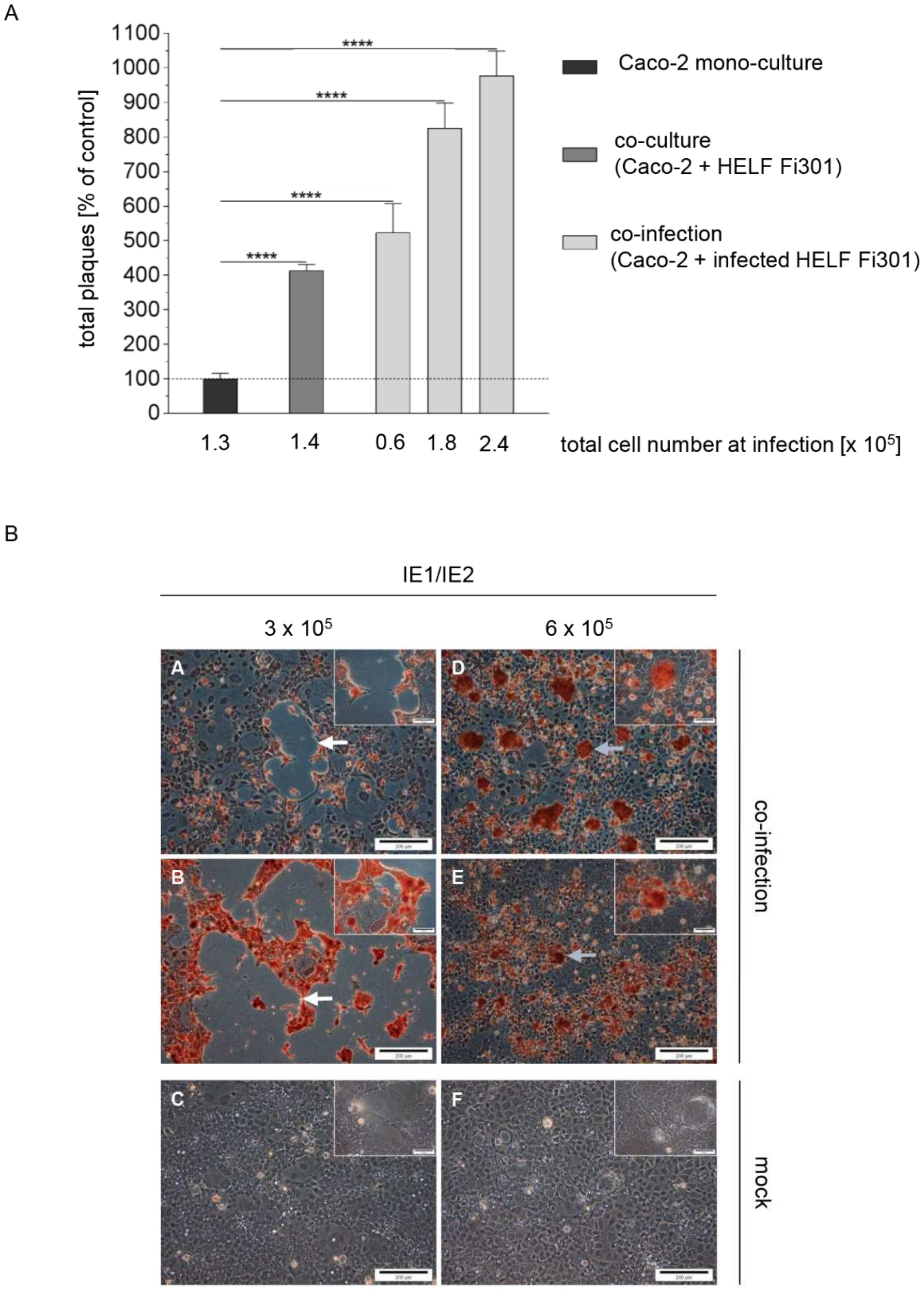

Figure S1. Effects of co-culture and co-infection on the HCMV infection of the Caco-2 cells. (A) Plaque reduction assay with Caco-2 mono-cultures and co-cultures infected as well as Caco-2 with HCMV-infected HELF Fi301 were performed at 7 d p.i. Total plaques [%] are normalized against those of the infected Caco-2 mono-culture (dashed line). The total cell count on infection, as determined by trypan blue exclusion test, was averaged from four experiments. Values represent mean ± SD from four independent experiments. (B) Analysis of the effect of co-infection by plaque reduction assay. Caco-2 cells were co-cultured with HCMV infected HELF Fi301 for 7 d p.i. Plaques were visualized using the AEC staining kit (A-B, D-E). Mock-infected cells served as controls (C, F). The bar represents 200 µM. White arrows: lysis of monolayers; grey arrows: infected proliferating cell. ****p<0.0001.
